# Tumor Cell “Slimming” Regulates Tumor Progression through PLCL1/UCP1‐Mediated Lipid Browning

**DOI:** 10.1002/advs.201801862

**Published:** 2019-03-25

**Authors:** Zhiyong Xiong, Wen Xiao, Lin Bao, Wei Xiong, Haibing Xiao, Yan Qu, Changfei Yuan, Hailong Ruan, Qi Cao, Keshan Wang, Zhengshuai Song, Cheng Wang, Wenjun Hu, Zeyuan Ru, Junwei Tong, Gong Cheng, Tianbo Xu, Xiangui Meng, Jian Shi, Zhixian Chen, Hongmei Yang, Ke Chen, Xiaoping Zhang

**Affiliations:** ^1^ Department of Urology Union Hospital Tongji Medical College Huazhong University of Science and Technology Wuhan 430022 China; ^2^ Institute of Urology Union Hospital Tongji Medical College Huazhong University of Science and Technology Wuhan 430022 China; ^3^ Department of Pathogenic Biology School of Basic Medicine Huazhong University of Science and Technology Wuhan 430030 China

**Keywords:** clear cell renal cell carcinoma, lipid browning, PLCL1, tumor cell “slimming”, UCP1

## Abstract

Emerging evidence has highlighted the important role of abnormal lipid accumulation in cancer development and progression, but the mechanism for this phenomenon remains unclear. Here, it is demonstrated that phospholipase C‐like 1/uncoupling protein 1 (PLCL1)/(UCP1)‐mediated lipid browning promotes tumor cell “slimming” and represses tumor progression. By screening three independent lipid metabolism‐related gene sets in clear cell renal cell carcinoma (ccRCC) and analyzing the TCGA database, it is found that PLCL1 predicted a poor prognosis and was downregulated in ccRCC. Restoration of PLCL1 expression in ccRCC cells significantly represses tumor progression and reduces abnormal lipid accumulation. Additionally, a phenomenon called tumor cell “slimming,” in which tumor cell volume is reduced and lipid droplets are transformed into tiny pieces, is observed. Further studies show that PLCL1 promotes tumor cell “slimming” and represses tumor progression through UCP1‐mediated lipid browning, which consumes lipids without producing ATP energy. Mechanistic investigations demonstrate that PLCL1 improves the protein stability of UCP1 by influencing the level of protein ubiquitination. Collectively, the data indicate that lipid browning mediated by PLCL1/UCP1 promotes tumor cell “slimming” and consumes abnormal lipid accumulation, which represses the progression of ccRCC. Tumor cell “slimming” offers a promising new concept and treatment modality against tumor development and progression.

## 1 Introduction

Tumors are often characterized by metabolic abnormalities, and lipid metabolism dysfunction is one of the most important types of abnormalities that should not be disregarded.^1^ Renal cell carcinoma (RCC) is one of the most common and lethal neoplasms in the urologic system.^2^ Among all RCC subtypes, clear cell renal cell carcinoma (ccRCC) is the most prevalent, constituting approximately 70–80% of RCC cases.^3^ ccRCC is known for metabolic disorders, the most significant of which involves lipid metabolism.^4^ It contains many lipid droplets (LD) consisting mainly of triglycerides and cholesterol‐esters.^5^, ^6^ Recent studies indicated that this abnormal lipid accumulation played an important role in the occurrence and progression of ccRCC. It can stabilize the endoplasmic reticulum, relieve endoplasmic reticulum stress, and has the ability to enhance cell viability to promote ccRCC progression.^7^ Nevertheless, mechanisms for the dispersal of abnormal lipids in ccRCC remain unclear.

Human adipose tissue can be divided into white adipose tissue (WAT) and brown adipose tissue (BAT); WAT is used to store energy, and BAT is used to produce heat.^8^ Lipid browning is the process by which WAT acquires the properties of BAT.^9^ It is a process that can promote the consumption of lipids through thermogenesis instead of adenosine triphosphate (ATP) synthesis.^10^ Uncoupling protein 1 (UCP1), one of the most important markers of BAT, resides within the inner membrane of mitochondria.^11^ It permits significant inner membrane proton conductance and uncouples cellular respiration from ATP synthesis ultimately generating heat.^12^ This process has been extensively researched for applications in obesity and diabetes, but it has never been directly studied in the lipid metabolism of tumors.

Phospholipase C‐like 1 (PLCL1) is a protein homologous to the PLC family; they have the similar structures, but PLCL1 lacks catalytic activity.^13^, ^14^ Mutations of the amino acids within the catalytic domain of PLCL1 make it impossible to convert PIP2 to inositol 1,4,5‐trisphosphate (IP3) and diacylglycerol (DAG).^15^ It can silence important PLC signaling downstream of Gq‐protein‐coupled receptors and cannot attenuate IP3‐dependent Ca2+ release from the endoplasmic reticulum (ER).^16^, ^17^ In terms of function, it always plays a role as a scaffolding protein to bind phosphatases 1 and 2A (PP1 and PP2A) as well as serine/threonine protein kinase (AKT), which relays intracellular signals downstream of phosphoinositide 3‐kinase (PI3K).^18^, ^19^ It can also regulate lipolysis in adipose tissue and correlate with the blood high density lipoprotein (HDL)‐cholesterol levels.^20^, ^21^ Each of these findings was obtained in non‐neoplastic diseases.

In this study, we found a new phenomenon called tumor “slimming” in which tumor cell volume was reduced and lipid droplets transformed into small pieces. Mechanistic and functional research showed that PLCL1 promoted this process and repressed tumor progression through lipid browning mediated by UCP1. Tumor “slimming” may provide new concepts and strategies for the treatment of ccRCC.

## 2 Results

### 2.1 PLCL1 Was Downregulated and Predicted Poor Prognosis in ccRCC

**Figure 1:**
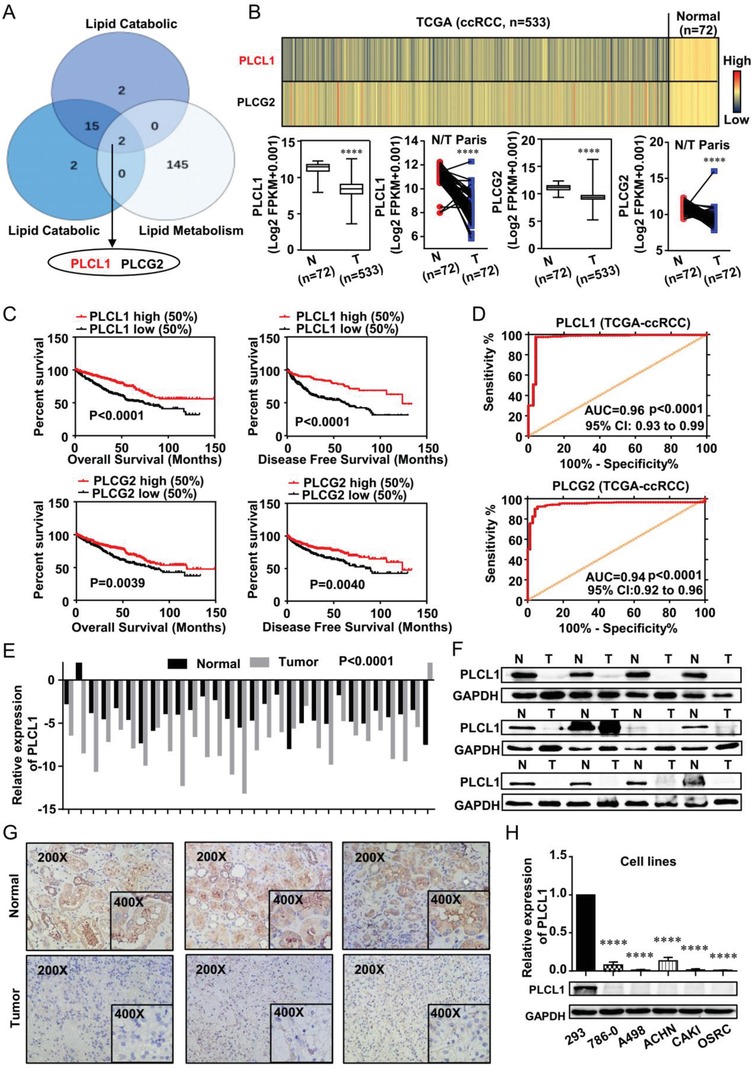
PLCL1 was downregulated and predicted poor prognosis in ccRCC. A) A Venn diagram of three independent lipid‐related gene sets from the Oncomine database (https://www.oncomine.org) and the European Bioinformatics Institute (EMBL‐EBI) (https://www.ebi.ac.uk). (All gene sets are subgene sets of differentially expressed genes in ccRCC.) B) The mRNA levels of PLCL1 and PLCG2 in 533 ccRCC tissues and 72 paired tissues in ccRCC based on data from the TCGA database. (In the color scheme of the heatmap, the colder color represents the lower gene expression level, and the warmer color represents the higher gene expression level.) *t*‐test, *p* < 0.0001. C) The Kaplan–Meier curves of PLCL1 and PLCG2 in ccRCC for both overall survival (OS) and disease‐free survival (DFS). D) The ROC (receiver operating characteristic) curves of PLCL1 (AUC = 0.9642 95% CI: 0.9343 to 0.9941; *p* < 0.0001) and PLCG2 (AUC = 0.9466 95% CI: 0.9253 to 0.9678; *p* < 0.0001) in ccRCC. E) The mRNA levels of PLCL1 in 30 ccRCC tissues and adjacent nonmalignant tissues. *t*‐test, *p* < 0.0001. F) The protein levels of PLCL1 in ccRCC tissues and adjacent nonmalignant tissues (Abbreviation: N, Normal tissue; T, Tumor tissue). G) The immunohistochemistry (IHC) staining for PLCL1 in ccRCC tissues and adjacent nonmalignant tissues (Magnification: 200× & 400×). H) The mRNA and protein levels in five ccRCC cell lines (786‐0, A498, ACHN, CAKI, and OSRC) and normal cell line (293). *t*‐test, ****p < 0.0001.

Previous studies have shown that lipid accumulation could promote the progression of ccRCC, but the mechanism of the consumption of these abnormal lipids was unknown.^7^ By screening three independent metabolic‐related subgene sets of differentially expressed genes in ccRCC by public databases, namely, a lipid metabolism‐associated gene set and two lipid catabolism downregulation‐related gene sets, two genes were found to show dysregulation in ccRCC (**Figure** **1**A). To assess the expression of these two genes in ccRCC, we performed analysis in the TCGA‐KIRC database that is composed of 533 ccRCC cases including 72 paired normal cases (the paired normal cases are derived from normal kidney tissue or adjacent renal tissue of the corresponding ccRCC patients as a control for ccRCC tissue). As shown in Figure 1B, the heatmap indicated that the color of the expression profiles of these two genes in ccRCC tissues was colder‐colored than normal tissues, which meant that the messenger RNA (mRNA) levels of these two genes were both significantly lower in ccRCC tissues compared with normal tissues, Box‐plots also showed similar results, and we found that the low expression of PLCL1 was more pronounced in ccRCC. Kaplan–Meier curves were measured to analyze whether survival time was associated with the expression of these two genes. The results showed that low levels of these two genes predicted a shorter survival time for overall survival (OS) and disease‐free survival (DFS) (Figure 1C). ROC (receiver operating characteristic) curves were then used to assess the ability to distinguish ccRCC patients from healthy individuals, and the curves indicated that both genes can identify ccRCC. PLCL1 had a relatively higher AUC (area under the curve), suggesting that it had a better diagnostic value for ccRCC than PLCG2 (Figure 1D). Comprehensive consideration based on these results placed our focus on PLCL1 for further study. To clarify the importance of PLCL1 in ccRCC, additional bioinformatic analyses were conducted. The downregulation of PLCL1 in ccRCC was further confirmed based on five additional data sets from the Oncomine database (Figure 1A, Supporting Information). Then, according to the in‐depth analysis of the TCGA database, we found that the expression of PLCL1 in ccRCC has a significant decreasing trend with the increase of ccRCC stage and grade (including T stage, N stage, nonmetastasis/metastasis, TNM stage, and G stage), thus, the PLCL1 expression is negatively correlated with tumor grade and stage (T stage, *p* < 0.0001, Spearman *r* = −0.232; N stage, *p* = 0.002, Spearman *r* = −0.193; nonmetastasis/metastasis, *p* < 0.0001, Spearman *r* = −0.170; TNM stage, *p* < 0.0001, Spearman *r* = −0.264; G stage, *p* < 0.0001, Spearman *r* = −0.244) (Figure 1B, Supporting Information) and that it was highly correlated with the clinicopathological parameters in ccRCC (**Table** **1**). Univariate and multivariate analyses were used to show that PLCL1 is an independent prognostic marker for ccRCC (**Tables** **2** and **3**).

**Table 1:** Correlation between PLCL1 mRNA expression and clinicopathological parameters of ccRCC patients

Parameter		Number	PLCL1 mRNA expression		*p* value
			Low (*n* = 258)	High (*n* = 259)	
Age (years)	< = 60	257	131	126	
	>60	260	127	133	0.660
Gender	Female	181	72	109	
	Male	336	186	150	0.001
T stage	T1+T2	332	141	191	
	T3+T4	185	117	68	0.000
N stage	N0+ NX	503	245	258	
	N1	14	13	1	0.001
M stage	M0+MX	441	205	236	
	M1	76	53	23	0.000
G stage	G1+G2	239	89	150	
	G3+G4	279	169	109	0.000
Stage	I+II	314	126	188	
	III+IV	203	132	71	0.000

**Table 2:** Univariate and multivariate analyses of PLCL1 mRNA level and patient survival

Variable	Univariate analysis			Multivariate analysisc)		
	HRa)	95% CIb)	P	HR	95% CI	P
Overall survival (*n* = 517)						
Age (years)						
≤60 (*n* = 257)	1.766	1.297–2.404	0.000	1.717	1 .258–2.343	0.001
>60 (*n* = 260)						
Gender						
Female (*n* = 181)	0.965	0.707–1.318	0.825			
Male (*n* = 336)						
T stage						
T1 or T2 (*n* = 332)	3.043	2.245–4.124	0.000	1.660	1.173–2.350	0.004
T3 or T4 (*n* = 185)						
N stage						
N0 or NX (*n* = 503)	3.554	1.871–6.748	0.000			
N1 (*n* = 14)						
M stage						
M0 or MX (*n* = 441)	4.369	3.197–5.971	0.000	2.940	2.070–4.173	0.000
M1 (*n* = 76)						
G grade						
G1 or G2 (*n* = 239)	2.605	1.853–3.661	0.000	1.606	1.118–2.307	0.010
G3 or G4 (*n* = 278)						
PLCL1						
Low (*n* = 258)	0.526	0.385–0.718	0.000	0.615	0. 449–0.844	0.003
High (*n* = 259)						

^a)^ Multivariate models were adjusted for T, N, M classification, age, and gender

^b)^ Hazard ratio, estimated from Cox proportional hazard regression model

^c)^ Confidence interval of the estimated HR.

**Table 3:** Univariate and multivariate analyses of PLCL1 mRNA level and patient survival

Variable	Univariate analysis			Multivariate analysisa)		
	HRb)	95% CIc)	P	HR	95% CI	P
Disease–free survival (*n* = 421)						
Age (years)						
≤60 (*n* = 228)	1.363	0.957–1.941	0.086			
>60 (*n* = 193)						
Gender						
Female (*n* = 142)	1.421	0.956–2.111	0.082			
Male (*n* = 279)						
T stage						
T1 or T2 (*n* = 282)	4.503	3.117–6.504	0.000	2.127	1.401–3.228	0.000
T3 or T4 (*n* = 139)						
N stage						
N0 or NX (*n* = 409)	5.915	2.969–11.781	0.000	2.768	1.358–5.639	0.005
N1 (*n* = 12)						
M stage						
M0 or MX (*n* = 370)	8.494	5.852–12.328	0.000	4.854	3.198–7.336	0.000
M1 (*n* = 51)						
G grade						
G1 or G2 (*n* = 207)	3.352	2.220–5.061	0.000	2.287	1.489–3.513	0.000
G3 or G4 (*n* = 214)						
PLCL1						
Low (*n* = 210)	0.449	0.308–0.654	0.000	0.674	0. 457–0.993	0.046
High (*n* = 211)						

^a)^ Multivariate models were adjusted for T, N, M classification, age, and gender

^b)^ Hazard ratio, estimated from Cox proportional hazard regression model

^c)^ Confidence interval of the estimated HR.

To further confirm the results from public databases, tumor tissues were extended to assess the mRNA and protein levels of PLCL1 in ccRCC. As shown in Figure 1E–G, PLCL1 mRNA and protein levels were significantly lower in ccRCC tissues than in normal tissues which were all obtained from the Department of Urology, Union Hospital, Tongji Medical College Wuhan, China. Furthermore, normal renal and ccRCC cell lines were also used to confirm the mRNA and protein levels of PLCL1. Similar to our previous results, we observed that all the ccRCC cells (786‐0, A498, ACHN, CAKI, OSRC) exhibited decreased expression of PLCL1 compared with the control cell line (293). (Figure 1H).

### 2.2 PLCL1 Repressed ccRCC Progression and Promoted Tumor Cell “Slimming” in ccRCC

**Figure 2:**
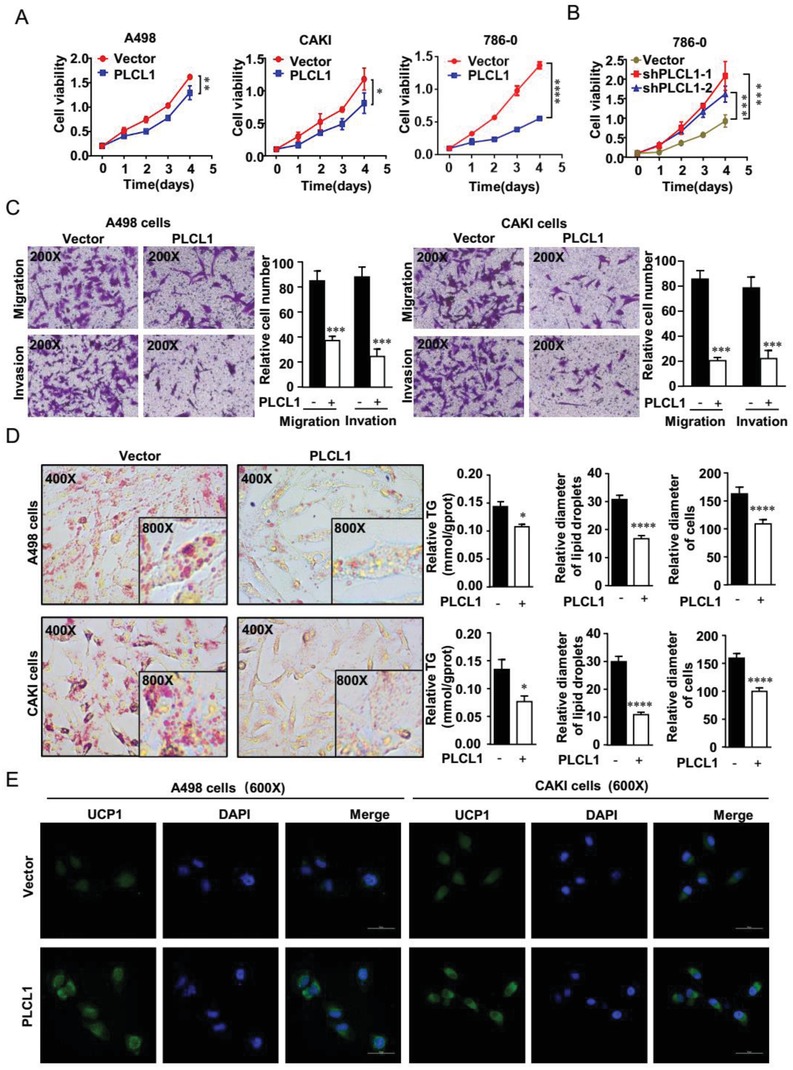
PLCL1 repressed ccRCC progression and promoted tumor cell “slimming” in ccRCC. PLCL1‐overexpressing or PLCL1‐knockdown ccRCC cell lines were constructed by transfecting overexpressing lentivirus and shRNA. The results were plotted as the mean ± SEM of three independent experiments with at least three replicates in each independent experiment. ****, *p* < 0.0001, ***, *p* < 0.001, **, *p* < 0.01, *, *p* < 0.05. A,B) Cell growth curves of CCK8 assays for indicated cells. C) Migration and invasion assay for indicated ccRCC cells (Magnification: 200×). D) Photomicrographs of Oil Red O staining of PLCL1‐overexpressing cell lines and the negative control (Magnification: 400× & 800×). Relative TG (mmol/gprot) tested by a triglyceride assay kit, relative diameters of lipid droplets, and relative diameters of cells for ccRCC cells described above. *t*‐test, ****, *p* < 0.0001, ***, *p* < 0.001, *, *p* < 0.05. E) Representative photographs of immunofluorescence of UCP1 for cells overexpressing PLCL1 (Magnification: 600×).

PLCL1 dysregulation in ccRCC suggested that PLCL1 may influence the progression of ccRCC. To test this hypothesis, we successfully constructed A498, CAKI, and 786‐0 cell lines with stably overexpressed PLCL1 by transfecting expression lentivirus and also constructed 786‐0 cell line with stably knocked down PLCL1 by transfecting short hairpin RNA (shRNA) (Figure 2A–D, Supporting Information). A CCK8 assay was used to assess the proliferation ability of ccRCC cells. The results showed that the proliferation rate of A498, CAKI, and 786‐0 cells was significantly repressed in cells overexpressing PLCL1 (**Figure** **2**A). Conversely, proliferation rates had increased in PLCL1 knockdown cells (Figure 2B). Likewise, the colony formation assay indicated similar results, thus, overexpression of PLCL1 inhibits the colony formation ability of cells and knockdown of PLCL1 increases the colony formation ability of cells (Figure 2E,G, Supporting Information). Transwell and wound healing assays were performed to assess migration and invasion. There was an obvious suppression of migration and invasion in PLCL1‐overexpressing cells (Figure 2C; Figure 2F,H,I, Supporting Information), while cells with stable PLCL1 knockdown showed the opposite result (Figure 2J,K, Supporting Information). Overall, PLCL1 could repress the progression of ccRCC.

**Figure 3:**
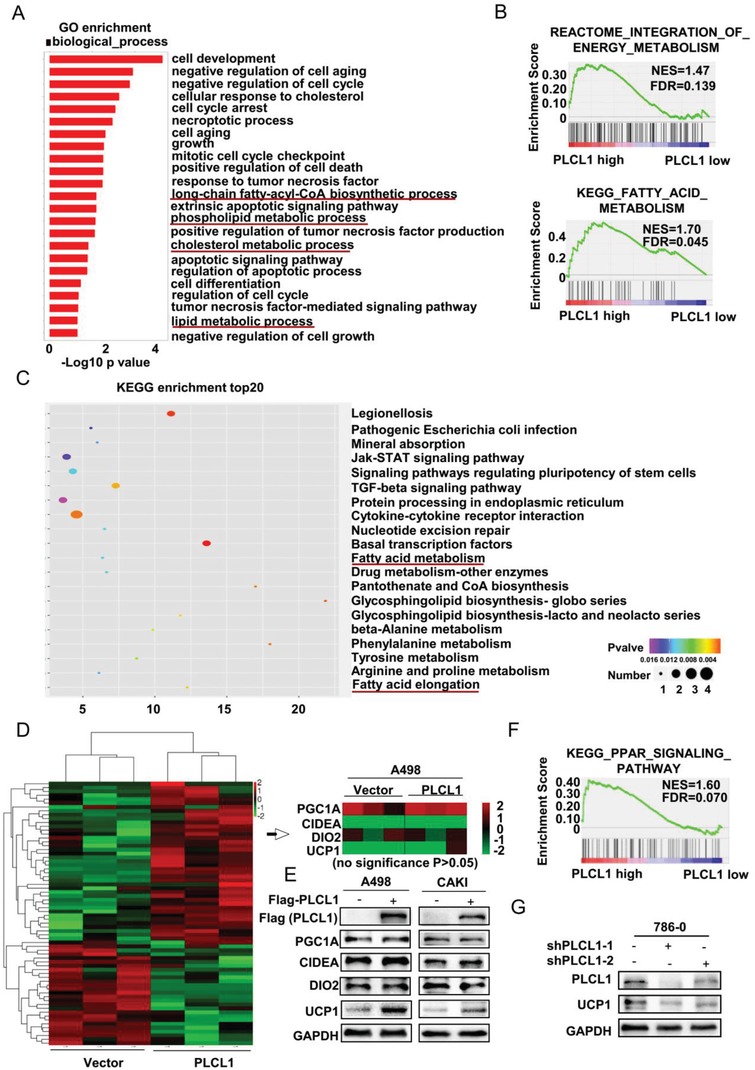
PLCL1 upregulated the expression of the lipid browning related gene UCP1 in ccRCC cells. Transcriptome sequencing was performed for A498 cells overexpressing PLCL1. A) GO enrichment for the indicated cells based on the results from sequencing. B) GSEA assays for the correlation of energy metabolism, fatty acid metabolism, and mRNA levels of PLCL1 according to the TCGA database. FDR < 25%, *p* < 0.05 was considered statistically significant. C) KEGG (Kyoto Encyclopedia of Genes and Genomes) enrichment top 20 for indicated cells based on the results from sequencing. D) The heatmap of cluster analysis based on sequencing results. E) Lipid browning‐related proteins were determined by western blot analysis. GAPDH was used as a loading control. F) GSEA assays for the correlation of PPAR signaling and mRNA level of PLCL1 according to TCGA database. FDR < 25%, p < 0.05 was considered as statistically significant. G) Western blot assay for the protein levels of PLCL1 and UCP1 in the PLCL1‐knockdown cells.

Previous studies have shown that abnormal lipid accumulation is one of the key factors in the progression of ccRCC. To explore the effect of PLCL1 on lipid accumulation, oil red staining and triglyceride analyses were used as a visual indicator of intracellular lipids in ccRCC. These results showed that there was an obvious lipid reduction and a significant triglyceride decrease in PLCL1 overexpression cells (Figure 2D; Figure 3A, Supporting Information), while the cells with stable PLCL1 knockdown showed the opposite result (Figure 3C, Supporting Information). In addition, an interesting phenomenon of reduced tumor cell volume and the transformation of lipid droplets into small pieces in PLCL1 overexpressed cells was observed, while the cells with stable PLCL1 knockdown showed the opposite result (Figure 2D; Figure 3B,D, Supporting Information). The consumption of lipids is accompanied by a decrease in tumor cell volume, which is why we called the new concept tumor cell “slimming.” On the macroscale, “slimming” is the ultimate goal of obesity treatment. Currently, one of the most important focuses and hotspots of obesity treatment is lipid browning.^22^, ^23^ Lipid browning could destroy energy homeostasis to consume lipids without producing ATP energy, and UCP1 is the most important marker of lipid browning.^24^ Immunofluorescence analysis and western blot showed that UCP1 was upregulated in cells stably overexpressing PLCL1 (Figure 2E; Figure 3E,F, Supporting Information), suggesting that lipid browning might take place in these cells. PLCL1 may promote tumor cell “slimming” by activating a reaction similar to lipid browning.

### 2.3 PLCL1 Upregulated the Expression of the Lipid Browning‐Related Gene UCP1 in ccRCC Cells

To clarify the mechanism of the lipid consumption mediated by PLCL1 more clearly, the A498 cells overexpressing PLCL1 were chosen to conduct whole transcriptome sequencing. Additional bioinformatic analyses were used to analyze the sequencing results. The results of gene ontology (GO) enrichment analysis showed that the functions of PLCL1 were not only related to basic cell growth, but also related to a variety of lipid metabolism processes, consistent with our findings above (**Figure** **3**A). Then, gene set enrichment analysis (GSEA) indicated that PLCL1 was highly associated with energy metabolism and fatty acid metabolism, both of which were related to the activation of lipid browning according to the data from TCGA database^8^ (Figure 3B). KEGG (Kyoto Encyclopedia of Genes and Genomes) enrichment analysis showed similar results (Figure 3C).

The basic process of lipid browning has been well studied, and each process involved has related key molecules. According to current authoritative studies on lipid browning, PGC1A, CIDEA, DIO2, and UCP1 are recognized as the four most important marker genes in lipid browning. They are widely involved in multiple processes of the lipid browning and can directly reflect changes in the various processes of lipid browning.^25^, ^26^ Therefore, we chose these four genes for subsequent studies of lipid browning. As the lipid browning was activated in stably overexpressed PLCL1 ccRCC cells, we created an mRNA expression heatmap of these four most critical browning‐related genes based on the results from sequencing to assess how the PLCL1 specifically affected the browning process. The results showed that there was no significant change in the mRNA levels of these four genes according to the heatmap (no significance *p* > 0.05) (Figure 3D). Nevertheless, the phenomenon of lipid browning was present in ccRCC cells according to our study. Thus, we used western blotting to analyze the protein levels of these four genes. The results showed that there were no significant changes in the expression of PGC1A. Meanwhile, CIDEA and DIO2 showed a slight increase after overexpressing PLCL1, and the expression of UCP1 was significantly increased in the cells overexpressing PLCL1 (Figure 3E). The expression changes of UCP1 were the most noticeable among all four browning‐related genes. Therefore, the activation of lipid browning in ccRCC cells overexpressing PLCL1 may be mediated by UCP1. Moreover, the GSEA analysis suggested that the function of PLCL1 was enriched in the peroxisome proliferators‐activated receptors (PPAR) pathway and could transactivate the PPRE (peroxisome proliferator response element) on the UCP1 enhancer to promote the expression of UCP1 based on the data from TCGA database^27^ (Figure 3F). These results provided further evidence that PLCL1 regulates UCP1. Similarly, the knockdown of PLCL1 in cells was accompanied by a decrease in UCP1 (Figure 3G). It could be concluded that PLCL1 promoted tumor cell “slimming” to consume the abnormal lipids, primarily by upregulating the expression of the lipid browning related gene UCP1.

### 2.4 UCP1 Repressed ccRCC Progression and Promoted Tumor Cell “Slimming” through Lipid Browning

**Figure 4:**
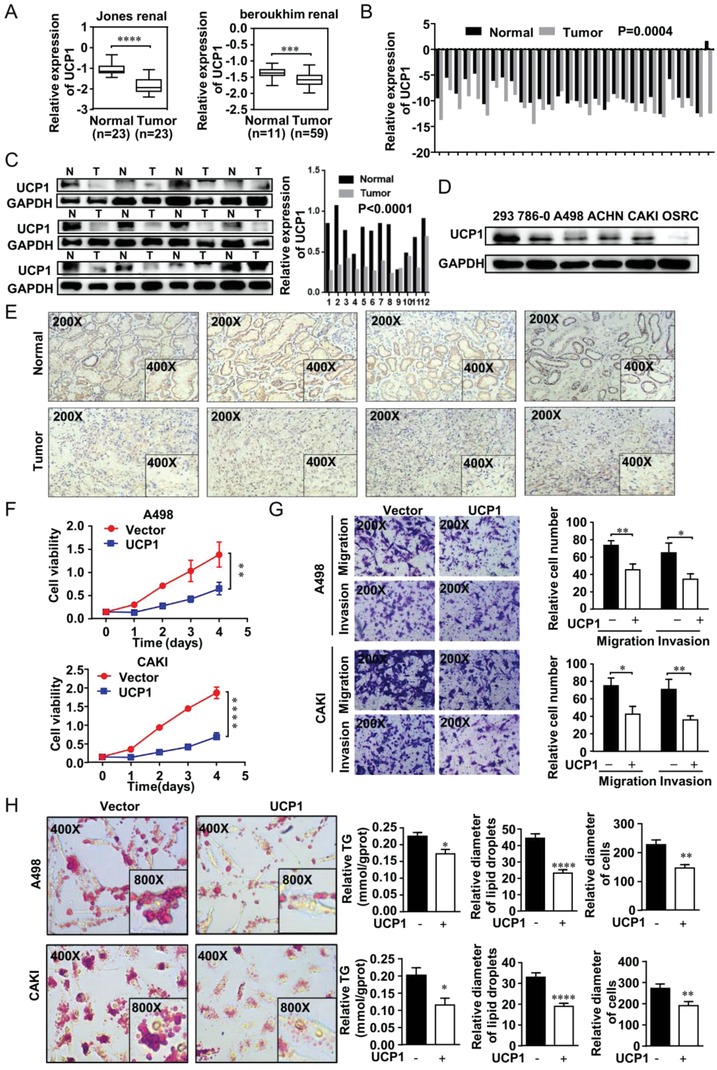
UCP1 repressed ccRCC progression and promoted tumor cell “slimming” through lipid browning. A) The mRNA of UCP1 in two independent gene sets from the Oncomine database (https://www.oncomine.org) in ccRCC. B) The mRNA levels of UCP1 in 30 ccRCC tissues and adjacent nonmalignant tissues. *t*‐test, *p* = 0.0004. C,D) The protein levels of UCP1 in ccRCC tissues and cell lines including five ccRCC cell lines (786‐0, A498, ACHN, CAKI, and OSRC) and normal cell line (293) (Abbreviation: N, Normal tissue; T, Tumor tissue). E) Immunohistochemistry (IHC) staining for PLCL1 in ccRCC tissues and adjacent nonmalignant tissues. F) UCP1‐overexpressing cell lines were constructed by plasmid transfection with both A498 and CAKI. Cell growth curves of CCK8 assays for indicated cells. ****, *p* < 0.0001, **, *p* < 0.01. G) Migration and invasion assay for indicated ccRCC cells (Magnification: 200×). *t*‐test, **, *p* < 0.01, *, *p* < 0.05. H) Photomicrographs of Oil Red O staining of overexpressed UCP1 cell lines and its negative control (Magnification: 400× & 800×). Relative TG (mmol/gprot) tested by triglyceride assay kit. Relative diameters of lipid droplets and relative diameters of cells in the ccRCC cells described above. *t*‐test, ****, *p* < 0.0001, ***, *p* < 0.001, *, *p* < 0.05.

As the most important marker of lipid browning, UCP1 is also a key molecule for initiating lipid browning.^28^ As shown in **Figure** **4**A–E, UCP1 mRNA and protein levels were significantly lower in ccRCC tissues than in normal tissues, and there was also a lower UCP1 expression in ccRCC cell lines (786‐0, A498, ACHN, CAKI, OSRC) compared with normal cell line (293). UCP1‐overexpressing A498 and CAKI cell lines were constructed by transfecting expression plasmids for UCP1 to activate lipid browning in ccRCC (Figure 4A, Supporting Information). A CCK8 assay showed that the lipid browning induced by UCP1 could significantly repress proliferation ability in ccRCC cells (Figure 4F). Moreover, a transwell assay showed that the migration and invasion ability were also obviously suppressed in cells overexpressing UCP1 (Figure 4G). From a lipid metabolism standpoint, UCP1‐overexpressing cells also showed a significant decrease in lipid accumulation accompanied by lipid droplet transformation into small pieces and a decrease in tumor cell volume (Figure 4H). Thus, the lipid browning activated by UCP1 could also promote tumor cell “slimming” in ccRCC.

### 2.5 PLCL1 Repressed ccRCC Progression and Promoted Tumor Cell “Slimming” through UCP1‐Mediated Lipid Browning

**Figure 5:**
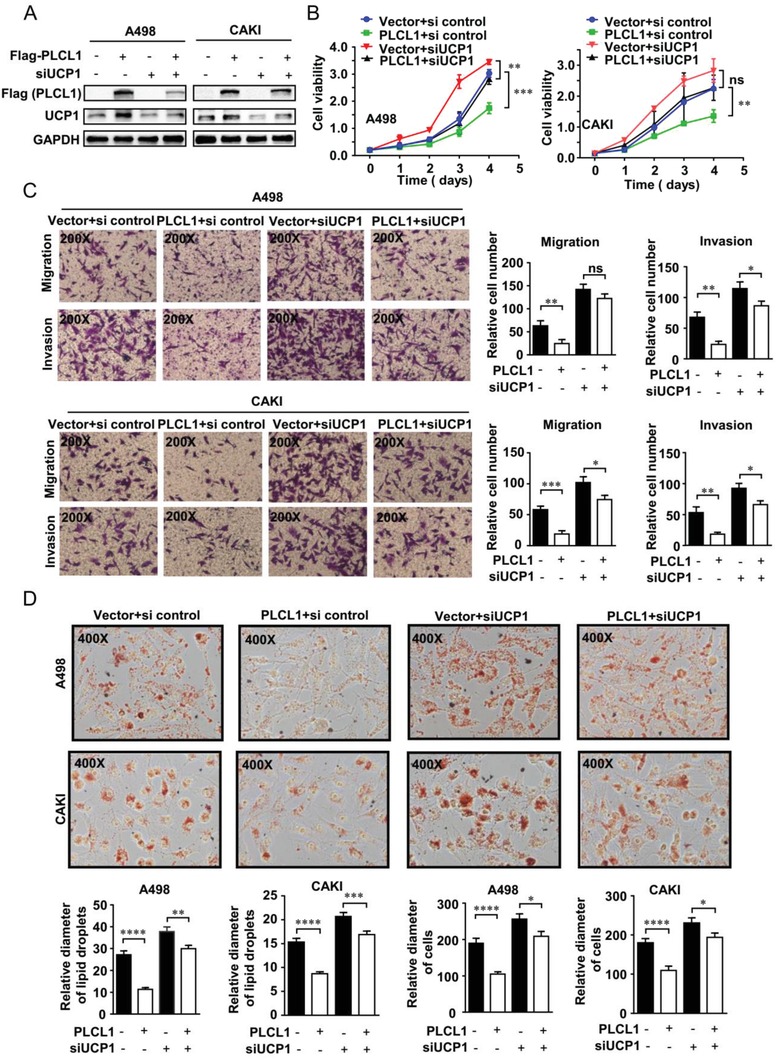
PLCL1 repressed ccRCC progression and promoted tumor cell “slimming” through UCP1‐mediated lipid browning. On the basis of PLCL1 overexpression, cells with UCP1 knocked down were constructed by transfecting UCP1 siRNA. We constructed four groups of cell lines for rescue experiments, including cell lines with PLCL1 expression lentivirus control vector and siUCP1 control, cell lines with PLCL1 expression lentivirus and siUCP1 control, cell lines with PLCL1 expression lentivirus control vector and siUCP1, and cell lines with PLCL1 expression lentivirus and siUCP1. A) Western blot assay for the protein levels of PLCL1 and UCP1 in indicated cells. B) Cell growth curves of CCK8 assays for indicated cells. ***, *p* < 0.001, **, *p* < 0.01, *p* = ns (no significance). C) Migration and invasion assay for indicated ccRCC cells (Magnification: 200×). *t*‐test, ***, *p* < 0.001, **, *p* < 0.01, *, *p* < 0.05, *p* = ns (no significance). D) Photomicrographs of Oil Red O staining of indicated cells (Magnification: 400×). Relative diameters of lipid droplets and the ccRCC cells described above.

To further illustrate the importance of the PLCL1‐UCP1 axis in ccRCC, functional rescue experiments were conducted. On the basis of overexpressing PLCL1, we constructed UCP1‐knockdown cells by transfecting UCP1 small interfering RNA (siRNA) (**Figure** **5**A). Through this method, we constructed four groups of cell lines for rescue experiments, including cell lines with PLCL1 expression lentivirus control vector and siUCP1 control, cell lines with PLCL1 expression lentivirus and siUCP1 control, cell lines with PLCL1 expression lentivirus control vector and siUCP1, and cell lines with PLCL1 expression lentivirus and siUCP1. At the same time, we also constructed cell lines with si control and cell lines with siUCP1 alone as a control. As previously mentioned, overexpressing PLCL1 could significantly decrease the proliferation ability of ccRCC cells. The CCK8 assay indicated that silencing UCP1 could attenuate the inhibition of cell proliferation induced by PLCL1 to a certain degree (Figure 5B; Figure 5A,B, Supporting Information). Then, the transwell assay was used to assess migration and invasion ability. Similar conclusions could be found in that silencing UCP1 could partly reverse the inhibition of migration and invasion induced by PLCL1 (Figure 5C). Subsequently, a consistent conclusion could also be drawn regarding its effects of lipid metabolism of ccRCC. Overexpressing PLCL1 alone resulted in a significant decrease in lipid accumulation. On this basis, silencing UCP1 could significantly increase the accumulation of lipids accompanied by the increase in lipid droplet diameter and cell diameter (Figure 5D). In other words, silencing UCP1 could largely reverse the biological effects induced by PLCL1. We conclude that PLCL1 repressed the progression of ccRCC and promoted tumor cell “slimming” mainly through UCP1‐mediated lipid browning.

### 2.6 PLCL1 Regulated the Protein Stability of UCP1 by Affecting UCP1 Ubiquitination Levels in ccRCC

**Figure 6:**
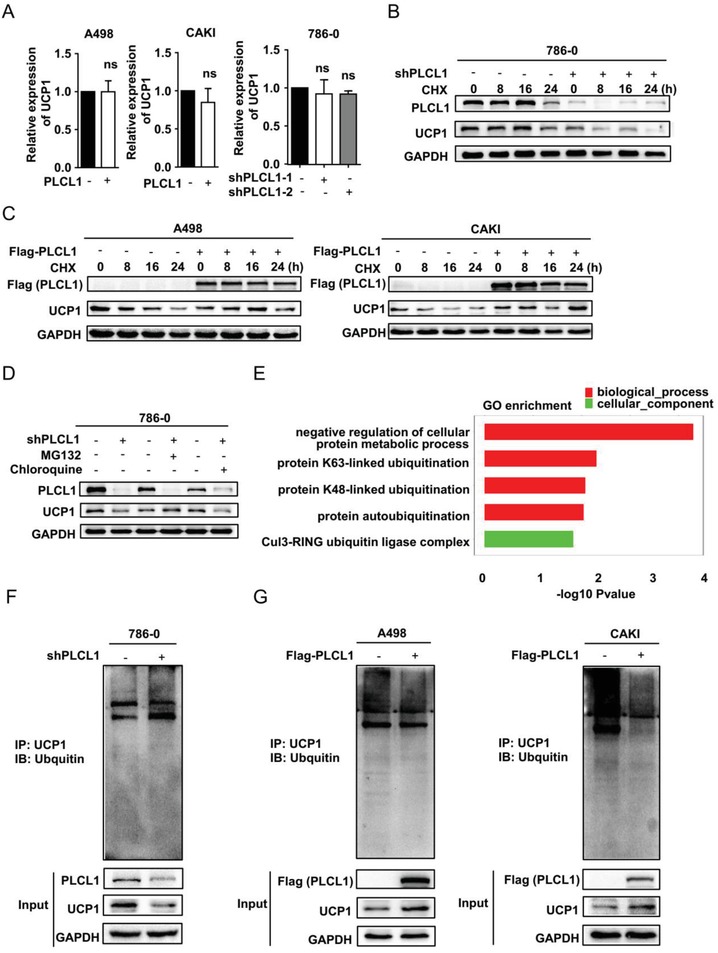
PLCL1 regulated the protein stability of UCP1 by affecting UCP1 ubiquitination levels in ccRCC. A) Relative mRNA levels of UCP1 in the cells PLCL1‐overexpressing and PLCL1‐knockdown cells. *p* = ns (no significance). B,C) ccRCC cells with stable PLCL1 knockdown and overexpression were treated with cycloheximide (CHX) at the indicated time points. Cells were collected, and UCP1 protein expression was analyzed by western blot. D) Cells with stable PLCL1 knockdown were treated with vehicle (DMSO), MG132 (20 × 10^−6^ m) or chloroquine (50 × 10^−6^ m) for 12 h. Western blotting was used to analyze the protein level of UCP1. E) GO enrichment for the indicated cells based on the results from sequencing. F,G) The cells with stable PLCL1 knockdown and overexpression were lysed and subjected to immunoprecipitation with an antibody against UCP1 and analyzed by western blotting with an anti‐ubiquitin antibody.

According to the description above, the results of whole transcriptome sequencing described in Figure 3 showed a phenomenon in which UCP1 protein levels were inconsistent with the mRNA levels. To further verify this phenomenon, we examined the mRNA level of UCP1 in ccRCC cells stably overexpressing PLCL1 and those with PLCL1 knocked down. The results showed no statistically significant changes in the mRNA levels of UCP1 (**Figure** **6**A). The inconsistency between protein levels and mRNA levels led us to consider whether PLCL1 directly affected the protein levels of UCP1. Protein stability regulation is an important form of post‐translational regulation that directly affects protein levels.^29^ To further investigate whether PLCL1 affected the stability of UCP1, control and PLCL1‐knockdown cells were treated with cycloheximide (CHX), an inhibitor of protein biosynthesis, for the indicated times. The results showed that there was a significant decrease over time in UCP1 protein levels in PLCL1‐knockdown cells and that the UCP1 protein levels were only slightly decreased after CHX treatment in control cells (Figure 6B). Similarly, the cells overexpressing PLCL1 were also treated with CHX. The cells significantly reduced the rate of decrease in UCP1 protein levels (Figure 6C). An overall conclusion could be drawn that PLCL1 enhanced the protein stability of UCP1 by inhibiting protein degradation.

The ubiquitin/proteasomal‐mediated pathway and the autophagy/lysosomal‐mediated pathway are important for mediating protein degradation in eukaryotic cells.^30^ To explore which pathway played a major role, a proteasome inhibitor (MG132) and a lysosome inhibitor (chloroquine) were administered to PLCL1‐knockdown cells. We found that only MG132 could reverse the decrease of UCP1 induced by PLCL1 (Figure 6D), which suggests that PLCL1 maintained the stability of UCP1 protein mainly by inhibiting protein degradation mediated by the ubiquitin/proteasomal pathway. GO enrichment analysis based on sequencing results also suggested that the functions of PLCL1 were enriched in several ubiquitin‐related pathways (Figure 6E). Then, ubiquitination‐related immunoprecipitation was used to assess the levels of ubiquitinated UCP1. The results showed that the level of ubiquitinated UCP1 was significantly increased in PLCL1‐knockdown cells (Figure 6F). Moreover, there was an obvious decrease in the level of ubiquitinated UCP1 in the cells overexpressing PLCL1 (Figure 6G). Collectively, we determined that PLCL1 regulated the protein stability of UCP1 by affecting UCP1 ubiquitination levels in ccRCC.

### 2.7 PLCL1 Repressed ccRCC Progression and Promoted Tumor Cell “Slimming” In Vivo

**Figure 7:**
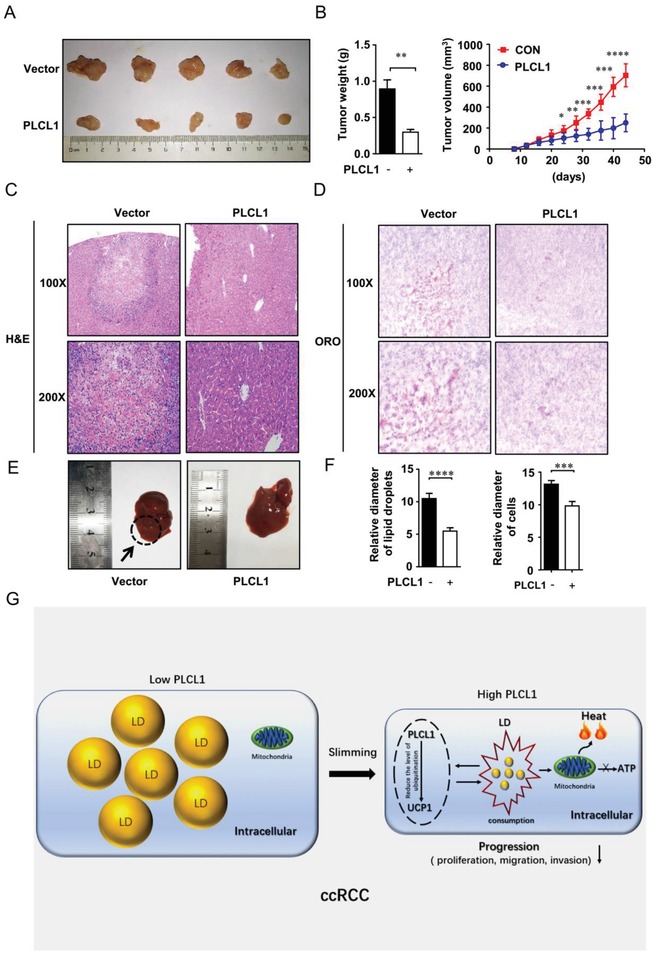
PLCL1 repressed ccRCC progression and promoted tumor cell “slimming” in vivo. A,B) CAKI cells stably overexpressing of PLCL1 were injected into nude mice. Tumor size was measured every four days. The data are shown as the mean ± SEM for separate tumors of each group. Images of tumors dissected from the mice. The tumor size (mm^3^) was plotted against days post‐tumor cell implantation. Tumors were weighted after resection at the end of experiment. C) Hematoxylin‐eosin (H&E) staining of the livers in the PLCL1‐overexpressing and control groups (Magnification: 100× & 200×). D) Photomicrographs of Oil Red O staining of the tumor xenograft in PLCL1 overexpressed and control groups (Magnification: 100× & 200×). *t*‐test, ****, *p* < 0.0001, ***, *p* < 0.001. E) Photomicrographs of the livers in the PLCL1‐overexpressing and control groups. F) Relative diameters of lipid droplets and cells for the tumor xenograft. G) Proposed model illustrating the protective function of PLCL1 and UCP1‐mediated lipid browning in ccRCC.

Encouraged by the observations described above, we assessed the role of PLCL1 in ccRCC in vivo. CAKI cells stably transduced with an overexpressed lentivirus of PLCL1 were implanted subcutaneously into nude mice. Tumor size was measured every four days, and the last measurement was performed on day 44. The results demonstrated that overexpression of PLCL1 could significantly reduce both the weight and the volume of tumors in vivo (**Figure** **7**A,B). IHC (immunohistochemistry) staining was conducted to evaluate the expression of PLCL1 and UCP1 in a xenograft tumor, and the results were consistent with the cell results. The high expression of PLCL1 was accompanied with increased levels of UCP1 (Figure 6A, Supporting Information). A nude mouse tail vein metastasis model was used to assess the metastatic ability of the tumor cells. We found that the group overexpressing PLCL1 significantly inhibited tumor liver metastases (Figure 7C,E). Oil red staining was then used to show the accumulation of lipid droplets in the xenograft tumor; the results showed that the group overexpressing PLCL1 had reduced lipid accumulation (Figure 7D). Statistics of lipid droplet size and cell diameter also displayed that overexpression of PLCL1 could achieve the purpose of tumor cell slimming by transforming the lipid droplets into tiny pieces, similar to the results in cells (Figure 7F).

Collectively, we suggest a model in which PLCL1 promotes tumor cell “slimming” by increasing the level of UCP1, allowing cells to consume lipids without producing ATP to repress the progression of ccRCC (Figure 7G).

## 3 Conclusion and Discussion

ccRCC is one of the most common tumors associated with dysfunctions in lipid metabolism, and the phenomenon of abnormal lipid accumulation in ccRCC has been observed for a long time.^4^ Previous studies have also demonstrated that abnormal LDs play a critical role in maintaining cellular homeostasis, which is a key factor in maintaining the viability of ccRCC cells and promoting disease progression.^7^ Nevertheless, strategies to treat these abnormal lipids remain unclear. In this study, we found an important lipid metabolism‐related gene called PLCL1 in ccRCC by bioinformatic analysis. Subsequent studies showed that PLCL1 could promote lipid browning by upregulating the protein level of UCP1, thereby promoting lipid consumption leading to tumor cell “slimming” and repressing tumor progression in ccRCC. Mechanistically, PLCL1 affects the protein level of UCP1 by reducing UCP1 ubiquitination, which can inhibit protein degradation and increase the stability of the UCP1 protein.

Metabolism is an intricate process that continuously maintains cellular dynamics. Once this balance is broken, the body will enter into an unhealthy state, leading to the occurrence of various metabolic diseases.^31^ The same conclusion can also be applied to lipid metabolism, which usually maintains a balance between synthesis and consumption.^32^ Abnormal lipid metabolism is the most common and intuitive. And it leads to one of the most prevalent diseases in humans called obesity.^33^ Tumors are also prominent in lipid metabolism, and ccRCC is one of the most typical types.^4^ Current research shows that the accumulation of lipids in ccRCC is due to the obstruction of the decomposition pathway, resulting in the synthesis of lipids being stronger than lipid decomposition, which is similar to the formation of obesity.^34^ It has been reported that obesity is an important risk factor for ccRCC.^35^, ^36^ These results suggest that obesity and ccRCC are inextricably linked. At the cellular level, the process of normal cells gradually accumulating lipids to transform into ccRCC cells is similar to the process of the body becoming morphologically obese. Therefore, we conclude that ccRCC in cells is analogous to obesity for the body.

Slimming is an inescapable topic in the field of obesity. Whether or not tumor cells could also be slimmed was the focus of this study. We found that lipid browning in ccRCC cells can be activated by overexpression of PLCL1. Lipid browning is currently the focus of research on the occurrence and treatment of obesity. Under normal circumstances, the decomposition of lipids is accompanied by the generation of ATP energy through the fatty acid beta‐oxidation pathway to maintain the balance of energy.^37^ However, this process allows the lipid to be consumed without producing ATP energy by the uncoupling of UCP1.^11^ The phenomenon of heat production without producing ATP causes the destruction of energy homeostasis, so energy consumption is relatively increased.^38^ Thus, a positive feedback that promotes lipid breakdown can be activated by increasing energy expenditure.^39^, ^40^ The consumption of lipids is a capability of the body and cells. For example, BAT can consume lipids more easily when WAT and BAT are stimulated by the same stimulus; this phenomenon is caused by UCP1‐mediated lipid browning.^41^ Previous studies have shown that UCP1 ablation can induce the occurrence of obesity and that upregulation of UCP1 can make it easier for the body to consume lipids under the same conditions to achieve the weight loss.^24^, ^42^, ^43^ Therefore, losing weight through lipid browning is a way to change the balance point of lipid metabolism, which can enable the body to achieve a new lipid metabolism balance in the direction of slimming rather than just improving lipid consumption.^44^

Since obesity is so similar to ccRCC, this phenomenon might also exist in ccRCC. Compared with normal cells, ccRCC cells do not show an accumulation of lipids as much as a decline in the ability to consume lipids. Studies have confirmed that UCP1 is downregulated in ccRCC, suggesting that the decline in the ability to consume lipids in obesity is highly likely to occur in ccRCC. The phenomenon we found is the upregulation of UCP1 protein levels by overexpressing PLCL1 to achieve tumor cell “slimming” through lipid browning. We suggest that this process changes the balance point of lipid metabolism in ccRCC cells and sets a new regulation point in the direction of lipid consumption and that the characteristics of this process for generating heat without producing ATP energy provides a basis for inhibiting the progression of tumors. It is well known that the growth of tumors requires a large amount of ATP energy^45^, ^46^ and that lipid browning not only does not provide the additional ATP required for tumor cell growth but also increases energy consumption, resulting in energy depletion to inhibit tumor growth in a similar manner to obesity.

This study is the first to discover and report the role of lipid browning in directly mediating lipid reduction in tumor cells. PLCL1 is found to be the key factor to regulate this process in ccRCC. The inspiration and excitement of this research are the discovery of this phenomenon. Whether this phenomenon can be extended to other tumors is the focus of our future studies. In conclusion, PLCL1 and activation of tumor cell “slimming” may provide new concepts and strategies for the treatment of ccRCC.

## 4 Experimental Section

*Cell Culture and Reagents*: The human renal cancer cell lines 786‐0, A498, ACHN, CAKI, OSRC, and the normal cell line HEK‐293 were purchased from The American Type Culture Collection (ATCC, USA). Cells were maintained in DMEM high glucose medium supplemented with 10% fetal bovine serum (FBS) and 1% penicillin–streptomycin and were cultured in a 5% CO_2_ incubator at 37 °C.

Expression lentivirus for PLCL1, shRNA for PLCL1, and corresponding control vector were all purchased from Genechem, China. Expression plasmids for PLCL1 (Ampicillin) and expression plasmids for UCP1 (Ampicillin) were also obtained from Genechem, China. siRNA for UCP1 (Target sequence: GTACAGAGCTAGTAACATA) came from Guangzhou Ribobio, China.

Protein synthesis inhibitor cycloheximide, proteasome inhibitor MG132, and autophagy inhibitor chloroquine were all obtained from MCE. Oil Red O (ORO) was provided by Wuhan Servicebio technology. Triglyceride assay kit was bought from Nanjing Jiancheng Bioengineering Institute.

*Cell Infection and Transfection*: Expression lentivirus for PLCL1 and corresponding control vector were transfected in A498, CAKI, and 786‐0 cell lines at an multiplicity of infection (MOI) of 30 using Polybrene (Genechem, China) and Enhanced Infection Solution (Genechem, China) according to the manufacturer's instructions from Genechem, China. shRNA for PLCL1 and corresponding control vector were transfected in 786‐0 cell line at an MOI of 45 using Polybrene (Genechem, China) and Enhanced Infection Solution (Genechem, China) according to the manufacturer's instructions from Genechem, China. Protein lysates and total RNA were collected 96 h after the transfection to verify transfection efficiency by western blot and qPCR. For plasmids transfections, A498 and CAKI cells were cultured in six‐well plants and transfected with 3 µg expression plasmids for PLCL1 or UCP1 using Lipofectamine 2000 (Invitrogen, CA, USA) according to the manufacturer's instructions respectively. siRNA for UCP1 was transfected in A498 and CAKI cell lines using Lipofectamine 2000 (Invitrogen, CA, USA) according to the manufacturer's instructions, respectively. Protein lysates and total RNA were collected 48 h after the transfection to verify transfection efficiency by western blot and qPCR.

*Tissue Samples*: 30 pairs of human ccRCC tissues and adjacent normal tissues were all obtained from the Department of Urology, Union Hospital, Tongji Medical College (Wuhan, China) during 2016–2017. They were at least 5 cm away from the tumor site in adjacent normal tissues. And they were stored in liquid nitrogen at −80 °C for RNA extraction. This process had fully informed consent of the patients. And this study was approved by the Institutional Review Board of Huazhong University of Science and Technology.

*Immunohistochemistry and Immunofluorescence Staining*: 4 µm formalin‐fixed paraffin‐embedded tissue sections were used for immunohistochemical staining. The sections were deparaffinized, rehydrated, and incubated in ethylenediaminetetraacetic acid (EDTA) at 120 °C for 5 min for antigen retrieval. After incubation with 3% H_2_O_2_ at room temperature for 15 min, fetal bovine serum was used to block the sections. Then, the slides were incubated with primary antibodies overnight at 4 °C (PLCL1 Abcam, ab157200, 1:150; UCP1 Abcam, ab10983, 1:100). Immunodetection was performed with 50 µl DAKO secondary antibody per section (Dako REAL EnVision Detection System, Peroxidase/DAB+, Rabbit/Mouse Code K5007) for 1 h at room temperature. Finally, DAB was used to visualize the immune complexes. And then, the sections were counterstained with hematoxylin.

For immunofluorescent staining, cells were fixed in 4% paraformaldehyde, permeabilized with 0.5% TritonX‐100 for 10 min, and blocked with 5% goat serum. And then, the cells were incubated with primary antibodies (UCP1 Abcam, ab10983, 1:250). Finally, Alexa Fluor 488‐conjugated Donkey Anti‐Rabbit IgG (H+L) (Abclonal, AS035, 1:250) was used as secondary antibody. And 4′,6‐diamidino‐2‐phenylindole (DAPI) was used to stain nuclei.

*RNA Isolation and Real‐Time PCR Analysis*: The TRizol reagent (Thermo, Massachusetts, USA) was used to extract total RNA of tissues and cells according to the manufacturer's instructions. The NanoDrop 2000 spectrophotometer (NanoDrop Technologies, Wilmington, USA) was used to measure the purity and concentration of the RNA solution. 1 µg of tissue or cell RNAs were applied for reverse transcription. qPCR analysis was conducted (LightCycler 480II; Roche, Basel, Switzerland) with the SYBR Green mix (Thermo, Massachusetts, USA). Samples were normalized by GAPDH.

GAPDH Forward 5′‐GAGTCAACGGATTTGGTCGT‐3′

Reverse 5′‐GACAAGCTTCCCGTTCTCAG‐3′

PLCL1 Forward 5′‐CGAAGCGTTGAACTCGATGT‐3′

Reverse 5′‐GAGCCATTACCTTCTGCTGC‐3′

UCP1 Forward 5′‐GCGGATGAAACTCTACAGCG‐3′

Reverse 5′‐TTGATTCCGTGGAGATGGCT‐3′

*Western Blotting Assays*: For western blotting assays, the protein of cells and tissues was extracted by radio‐immunoprecipitation assay (RIPA) protein lysis buffer (Beyotime Institute of Biotechnology, Haimen, China) with freshly added protease inhibitor cocktail and PMSF. 40 µg of protein was subjected to SDS‐PAGE gel. The proteins were then separated by gel electrophoresis and transferred to polyvinylidene fluoride (PVDF) membranes. 5% nonfat dried skimmed milk was used to block the membranes for 1 h at room temperature. Then, the membranes were incubated overnight with primary antibodies. Finally, the membranes were washed and incubated in blocking buffer with secondary antibodies (HRP‐conjugated Affinipure Goat Anti‐Mouse IgG(H+L), Proteintech, SA00001‐1, 1:2500; HRP‐conjugated Affinipure Goat Anti‐Rabbit IgG(H+L), Proteintech, SA00001‐2, 1:2500) for 2 h before detection. The antibodies used for western blots were: PLCL1 (Abcam, ab157200, 1:1000), UCP1 (Abcam, ab10983, 1:1000), Flag (Abclonal, AE005, 1:1000), GAPDH (Proteintech, 60004‐1‐Ig, 1:1000), PGC1A (Abclonal, A12348, 1:1000), CIDEA (Santa Cruz, sc‐8730‐R, 1:200), Ub (Santa Cruz, sc‐8017, 1:200), and DIO2 (Proteintech, 26513‐1‐AP, 1:1000).

*Whole Transcriptome Sequencing*: Total RNA was extracted using the mirVana miRNA Isolation Kit (Ambion) following the manufacturer's protocol. RNA integrity was evaluated using the Agilent 2100 Bioanalyzer (Agilent Technologies, Santa Clara, CA, USA). The samples with RNA Integrity Number (RIN) ≥ 7 were subjected to the subsequent analysis. The libraries were constructed using TruSeq Stranded mRNA LTSample Prep Kit (Illumina, San Diego, CA, USA) according to the manufacturer' s instructions. Then, these libraries were sequenced on the Illumina sequencing platform (HiSeq 2500 or Illumina HiSeq X Ten) and 125 bp/150 bp paired‐end reads were generated. Techniques and methods for whole transcriptome sequencing were provided by Oebiotech, China.

*Co‐Immunoprecipitation*: Ice‐cold phosphate buffer saline (PBS) was used to wash cells. And Triton‐lysis buffer was used to lyse the cells. The lysates were centrifuged at 12 000 × *g* for 10 min at 4 °C. Then, supernatant was precleared with 20 µL Protein A/G PLUS‐Agarose (Santa Cruz, CA USA) for 1 h at 4 °C and incubated with the appropriate antibody overnight at 4 °C, followed by precipitation with protein A/G PLUS‐Agarose. Finally, the immunoprecipitates were collected by washing and centrifugation for three times and boiled in 2 × SDS sample buffer before western blotting assay.

*Cell Viability Assays*: For cell viability assay, 2 × 10^3^ cells were plated in 96‐well plates. The proliferation rate of cells was detected using the CCK8 method based on the manufacturer's instructions. Cell viability was assessed at 0, 24, 48, 72 and 96 h upon treatments, respectively.

*Colony Formation Assays*: 1000 cells were plated into six‐well plates after 14 days to evaluate the effects of colony formation. Two weeks after cell seeding, surviving colonies (>50 cells per colony) were visualized with 0.05% crystal violet staining.

*Wound Healing Assays*: Cells were grown in six‐well plates. When cells reached 70–80% confluence, they were wounded by a 10 µl pipette tip through the monolayer. Then, cells were washed to remove cell debris and allowed to migrate for indicated hours. Images were taken at time 0, 12, 24 h / 0, 16, 32 h post wounding.

*Transwell Assays*: For migratory and invasion assays, cells were incubated in serum‐free medium for 24 h. Then, cells were seeded in the top chamber of the insert and cells were allowed to invade through the Matrigel. After 24 h, cells invading the lower surface of the membrane insert were fixed in 100% methanol. Then, cells were stained with 0.05% crystal violet and randomly chose 10 fields for counting.

*In Vivo Tumor Implantation*: A total of 2 × 10^6^ cells were injected subcutaneously into six week old male nude mice purchased from Vital River Laboratory Animal Technology Co. Ltd. And Nude mouse tail vein metastasis model was used to assess the metastatic ability of the tumor cells. A digital caliper was used to access the tumor growth every four days for seven weeks. Tumor weight was measured when mice were sacrificed on day 44 after cell implantation. Immunohistochemical staining under the standard procedure was conducted as previously described. All animal experiments were performed in accordance with animal protocols approved by the Institutional Animal Use and Care Committee of Tongji Medical College, Huazhong University of Science and Technology (S175).

*Bioinformatics Analysis*: Three independent lipid‐related gene sets from Oncomine database (https://www.oncomine.org) and the European Bioinformatics Institute (EMBL‐EBI) (https://www.ebi.ac.uk) were used for screening. The mRNA levels of genes in ccRCC patients, normal kidney tissues and clinical data about gender, age, recurrence, metastasis, TNM stage, overall survival, disease‐free survival of patients in TCGA‐KIRC Database were obtained from the cBioPortal (http://www.cbioportal.org/public‐porta) which includes mRNA profiles for 533 ccRCC cases including 72 paired normal cases (the paired normal cases were derived from normal kidney tissue or adjacent renal tissue of the corresponding ccRCC patients as a control for ccRCC tissue). The gene set enrichment analysis was used to assess pathways enriched in the gene set based on the pathway Enrichment Score (ES).

*Statistical Analysis*: All statistical analyses were performed using Excel 2016 (Microsoft) and SPSS Statistics 22.0 (IBM SPSS, Chicago, IL). All experiments in vitro were performed in triplicate and all data were represented as mean ± SEM. Statistical analyses were conducted using the Student's *t*‐test and Pearson correlation coefficient. The significance value was determined when *p* < 0.05.

## Conflict of Interest

The authors declare no conflict of interest.

## Supporting information

Supplementary
