# Supplementary material for: Tumor Cell “Slimming” Regulates Tumor Progression through PLCL1/UCP1‐Mediated Lipid Browning
Source: Adv Sci (Weinh). 2019 Mar 25;6(10):1801862. doi: 10.1002/advs.201801862 (PMC6523368; doi:10.1002/advs.201801862)

Copyright WILEY-VCH Verlag GmbH & Co. KGaA, 69469 Weinheim, Germany, 2019.

ADVANCED SCIENCE
Open Access

Supporting Information

for *Adv. Sci.,* DOI: 10.1002/advs.201801862

Tumor Cell "Slimming" Regulates Tumor Progression through PLCL1/UCP1-Mediated Lipid Browning

Zhiyong Xiong, Wen Xiao, Lin Bao, Wei Xiong, Haibing Xiao, Yan Qu, Changfei Yuan, Hailong Ruan, Qi Cao, Keshan Wang, Zhengshuai Song, Cheng Wang, Wenjun Hu, Zeyuan Ru, Junwei Tong, Gong Cheng, Tianbo Xu, Xiangui Meng, Jian Shi, Zhixian Chen, Hongmei Yang, Ke Chen, and Xiaoping Zhang**

Supplementary figure legends

Supplemental Fig 1 PLCL1 expression positively correlated with tumor grade and stage. (A) The mRNA of PLCL1 in five independent gene sets from the Oncomine database (https://www.oncomine.org) in ccRCC. (B) The mRNA level of PLCL1 in ccRCC patients among different subgroups including T stage, N stage, TNM stage, non-metastasis / metastasis and G stage (T stage, P<0.0001, Spearman r= -0.232; N stage, P=0.002, Spearman r= -0.193; non-metastasis / metastasis, P<0.0001, Spearman r= -0.170; TNM stage, P<0.0001, Spearman r= -0.264; G stage, P<0.0001, Spearman r= -0.244). A t-test was used to analyze the data of the two groups, and ANOVA analysis was used for the data with four groups. ****, p<0.0001, ***, p<0.001, **, p<0.01, *, p<0.05, P= ns (no significance).

Supplemental Fig 2 PLCL1 repressed ccRCC progression (proliferation, migration, and invasion). (A-D) PLCL1-overexpressing or PLCL1-knockdown ccRCC cell lines were constructed. Western blotting and qPCR were used to measure the protein level and mRNA level of PLCL1 for the cells indicated. t-test, ****, p<0.0001, ***, p<0.001, **, p<0.01, *, p<0.05 (E)A498 and CAKI cells with stably overexpressing PLCL1 were analyzed for growth by a colony formation assay. (F) A498 and CAKI cells with stably overexpressing PLCL1 were analyzed for migration by a wound healing assay. (G) Cells with PLCL1 knocked down were analyzed for growth by a colony formation assay. (H) 786-0 cells with stably overexpressing PLCL1 were analyzed for migration by a wound healing assay. (I) 786-0 cells with stably overexpressing PLCL1 were analyzed for migration and invasion by transwell assay (Magnification: 200X). (J, K) Cells with PLCL1 knocked down were analyzed for migration and invasion by a wound healing assay and transwell assay (Magnification: 200X).

Supplemental Fig 3 Lipid changes in 786-0 cell with PLCL1 overexpressed and knocked down. (A) Photomicrographs of the Oil Red O staining of 786-0 cell line with PLCL1 overexpressed (Magnification: 400X). (B) Relative TG (mmol/gprot) tested by a triglyceride assay kit. The relative

diameters of lipid droplets and the relative diameters of cells in 786-0 cell with PLCL1 overexpressed. t-test, ****, $p<0.0001$, ***, $p<0.001$, **, $p<0.01$, *, $p<0.05$. (C) Photomicrographs of the Oil Red O staining of PLCL1-knockdown cells (Magnification: 400X). (D) Relative TG (mmol/gprot) tested by a triglyceride assay kit. The relative diameters of lipid droplets and the relative diameters of cells in PLCL1-knockdown cells. t-test, ****, $p<0.0001$, ***, $p<0.001$, **, $p<0.01$, *, $p<0.05$. (E, F) UCP1 protein level in overexpressed PLCL1 cells measured by western blot.

Supplemental Fig 4 UCP1-overexpressing cells were constructed. (A) UCP1-overexpressing cells were constructed by plasmid transfection. The protein level of UCP1 was measured in the indicated cells by western blot.

Supplemental Fig 5 Effect of UCP1 knocked down on cell proliferation.

(A) The cells with UCP1 knocked down were constructed by siRNA transfection. The protein level and the mRNA level of UCP1 was measured in the indicated cells by western blot and qPCR. (B) Cell growth curves of CCK8 assays for indicated cells.

Supplemental Fig 6 Immunohistochemistry (IHC) staining for PLCL1 and UCP1 in the tumor xenograft. (A) Immunohistochemistry (IHC) staining for PLCL1 and UCP1 in the tumor xenograft (Magnification: 200X & 400X).

Supplementary Figure. 1

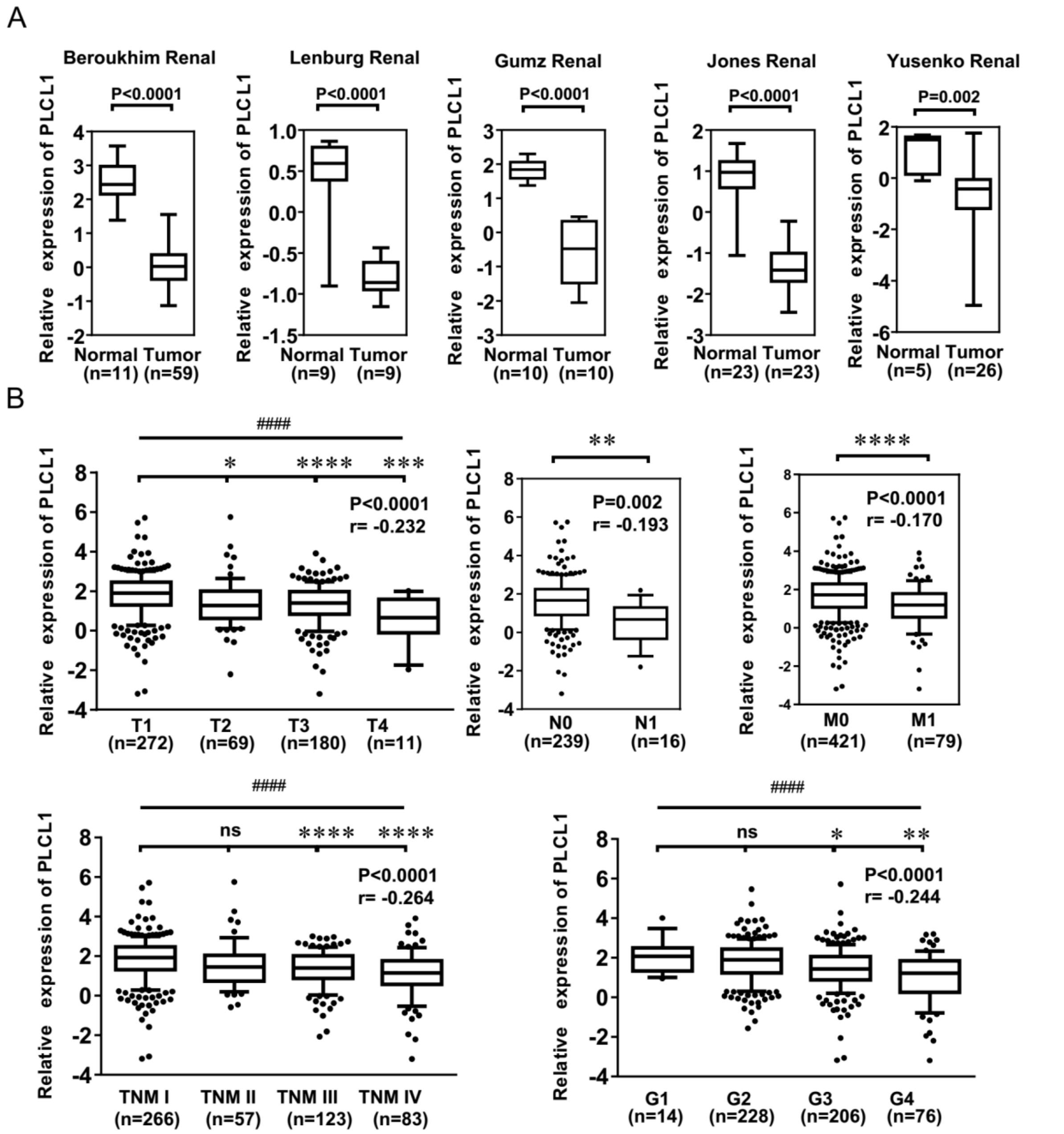

Supplementary Figure. 2

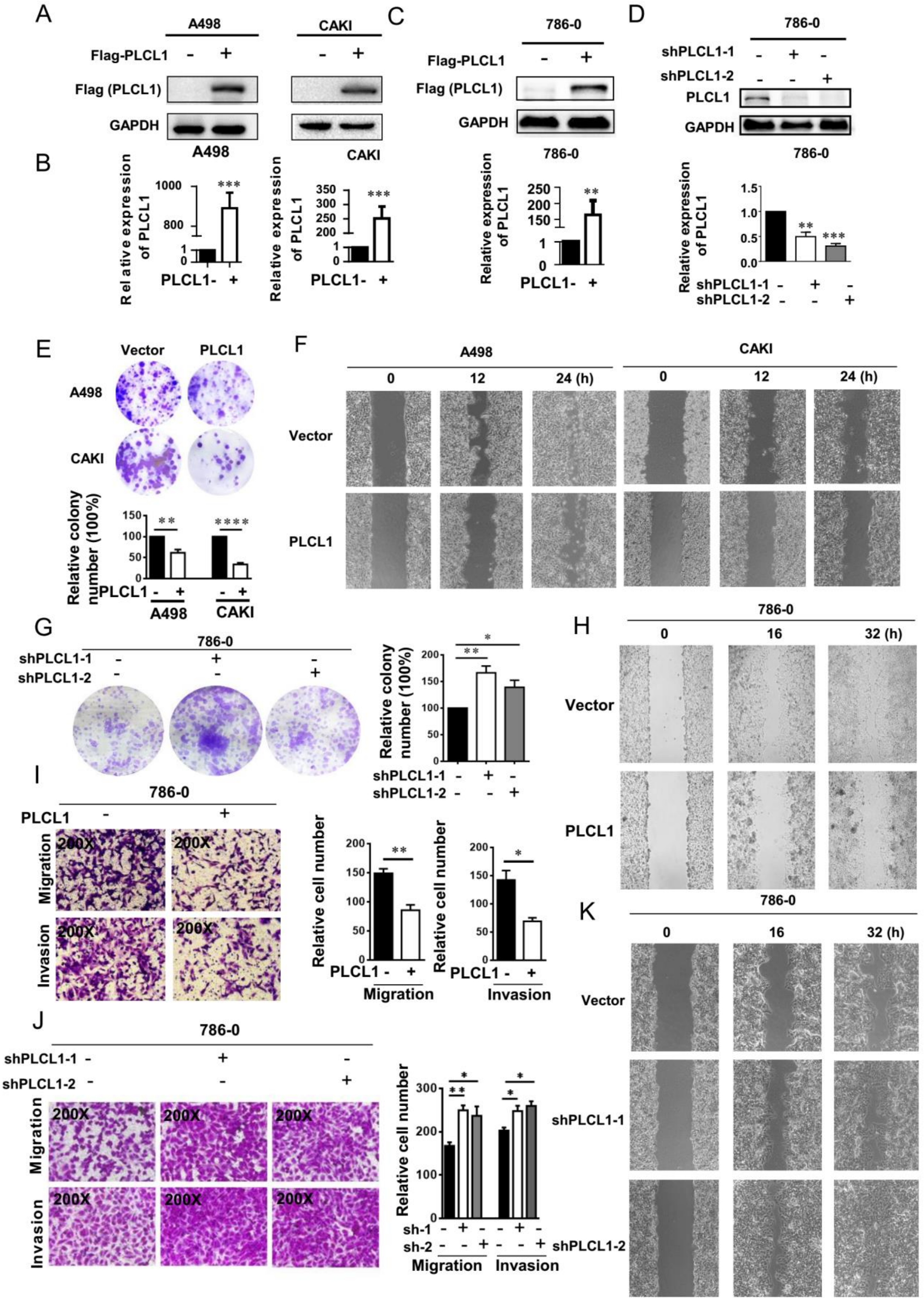

Supplementary Figure. 3

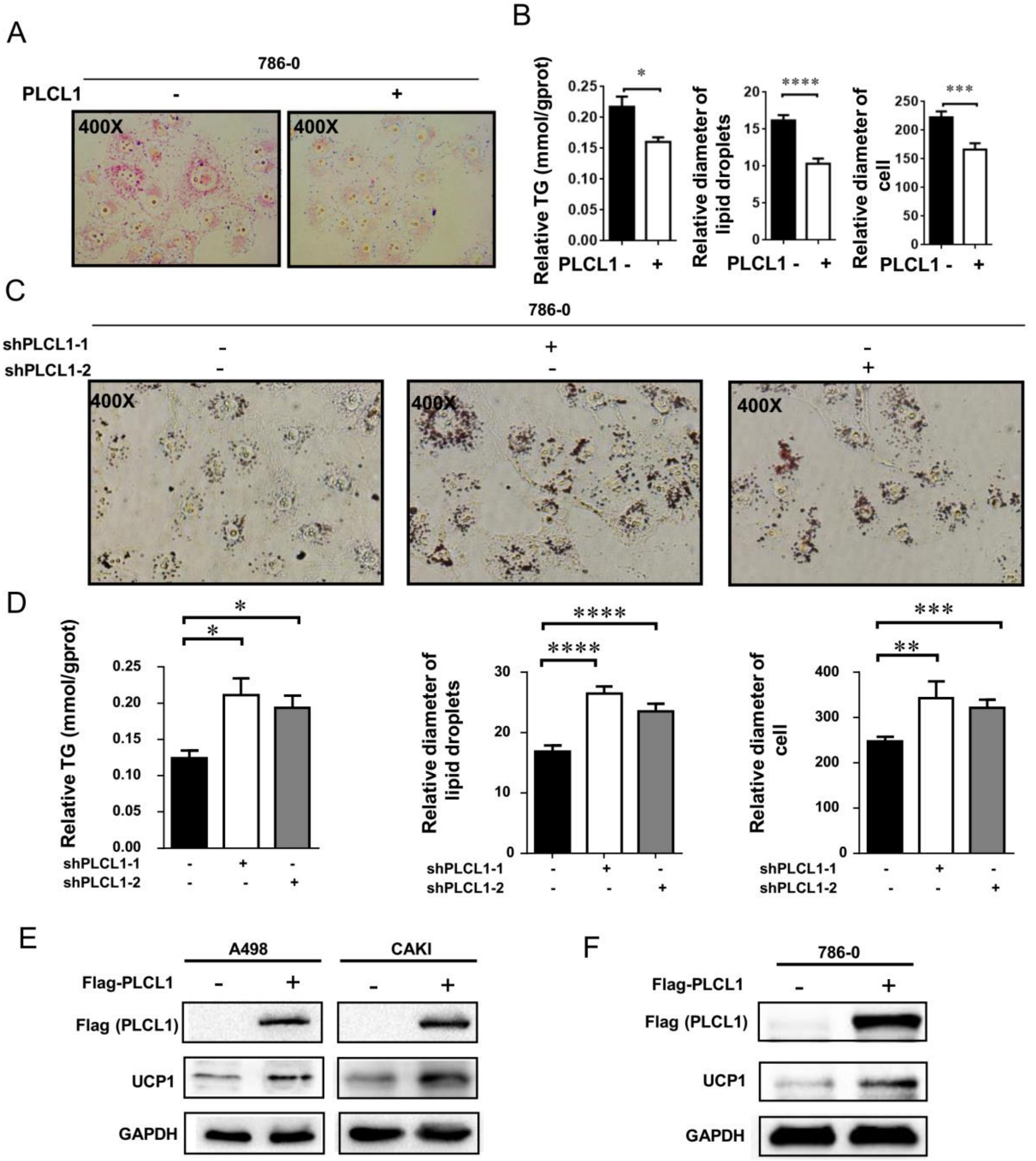

Supplementary Figure. 4

A

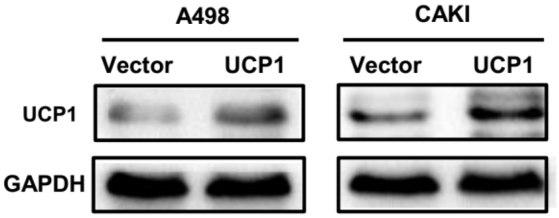

Supplementary Figure. 5

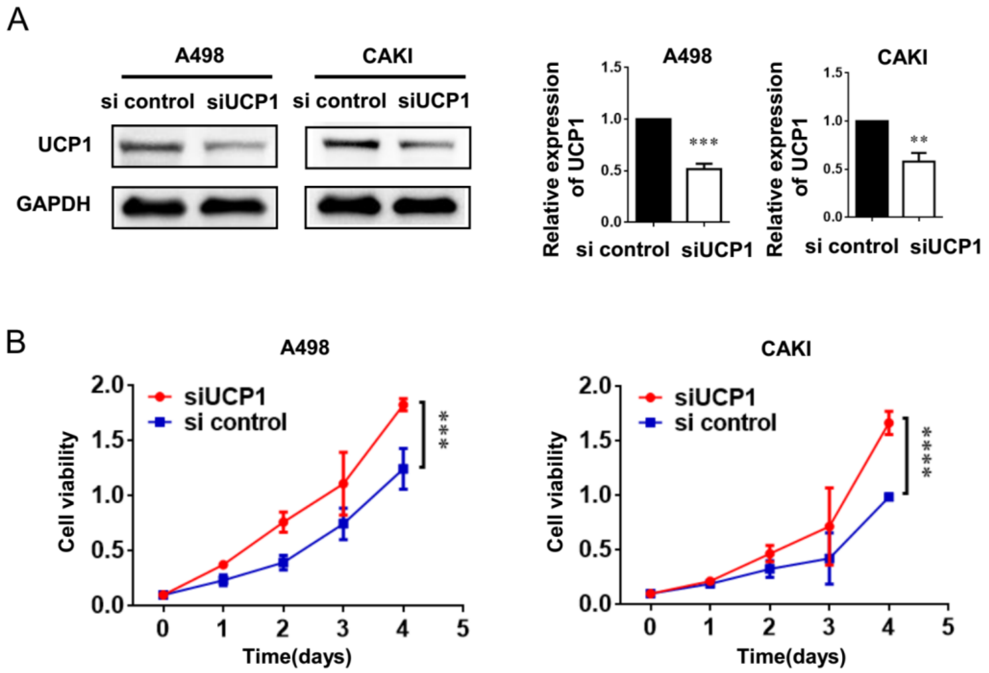

Supplementary Figure. 6

A

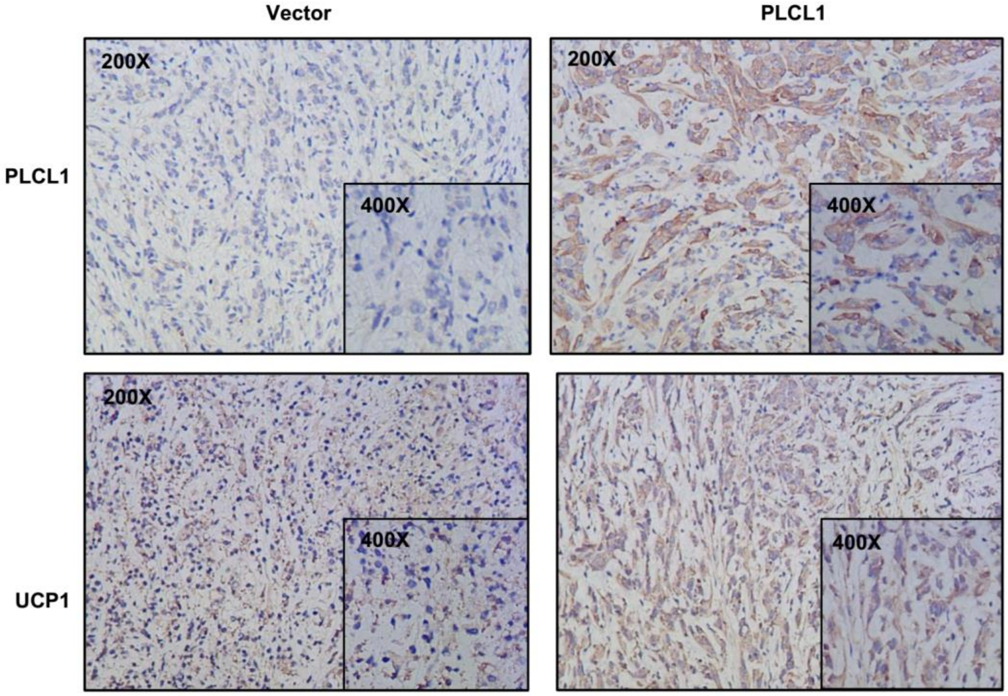

Supplement: Supplementary file 1 — Supplementary [file ADVS-6-1801862-s001.pdf]
